# Research on the degradation mechanism, product effects and optimization strategy of the tributyl phosphate solvent system in the PUREX process

**DOI:** 10.1002/smo2.70034

**Published:** 2026-01-05

**Authors:** Tian Lan, Jiaxin Liu, Yi Liu

**Affiliations:** ^1^ State Key Laboratory of Fine Chemicals, Frontiers Science Center for Smart Materials School of Chemical Engineering Dalian University of Technology Dalian China; ^2^ China Nuclear Power Engineering Co., Ltd. Beijing China

**Keywords:** degradation products, PUREX process, solvent degradation, third‐phase formation, tributyl phosphate (TBP)

## Abstract

While nuclear energy represents a low‐carbon and high‐efficiency energy source that plays a vital role in the global energy mix, the limitations of spent fuel reprocessing technology pose a major challenge to its sustainable development. The PUREX (plutonium uranium redox extraction) process is currently the dominant nuclear fuel reprocessing technology in the world. However, the key extractant in this process is tributyl phosphate (TBP), which degrades under intense radiation, high temperatures, and strong acidity. This leads to the production of dibutyl phosphate, monobutyl phosphate, and other degradation byproducts, which may reduce the extraction efficiency and trigger third‐phase formation and equipment corrosion. This paper systematically reviews the degradation mechanisms of TBP and its diluents, the analytical technique suitable for characterizing degradation products, and the impact of degradation products on the post‐treatment process. Additionally, optimization strategies employed for suppressing third‐phase formation are discussed. This study offers a theoretical foundation and technical insights in optimizing the PUREX process and ensuring the safe operation of the post‐treatment process.

## Graphical abstract

This paper focuses on reviewing the degradation mechanisms of tributyl phosphate and its diluents, the analytical technique suitable for characterizing degradation products, and the impact of degradation products on the post‐treatment process, offering a theoretical foundation and technical insights in optimizing the plutonium uranium redox extraction process and ensuring the safe operation of the post‐treatment process.

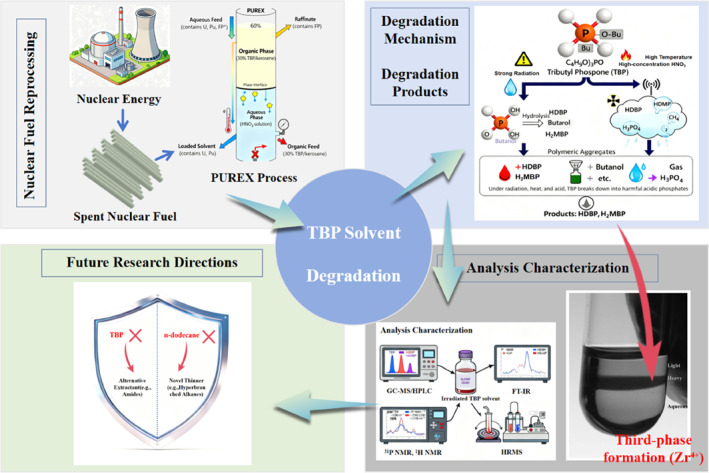

## 1 INTRODUCTION

**FIGURE 1:**
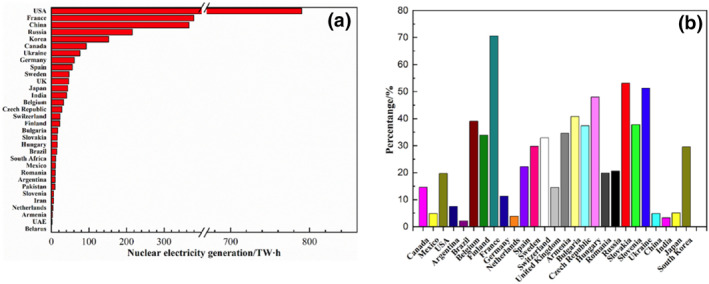
(a) Nuclear power generation and (b) proportion of nuclear power generation in total power generation in various countries.^[1]^

Nuclear energy represents a low‐carbon and high‐efficiency power source that plays a crucial role in the global energy landscape. Notably, the nuclear power sector in China has rapidly expanded (Figure 1). According to data from the International Atomic Energy Agency, in 2023, China's nuclear power generation reached 406.5 TW·h, with nuclear energy accounting for 4.9% of the total electricity output.^[2]^ Owing to its ability to reduce carbon emissions, the growth of the nuclear energy sector shows great promise for the generation of electricity.[^3^, ^4^] However, with increasing nuclear energy sector, increasing amounts of spent fuel were generated simultaneously. Therefore, rational disposal of the above fuel has emerged as a critical challenge for the nuclear energy industry.^[5]^

At present, spent fuel cycle methods are primarily classified into the Once‐Through Fuel Cycle (OTC) and the Closed Fuel Cycle (CFC).^[6]^ The OTC method does not involve any reprocessing methods, and the spent fuel is directly disposed as radioactive waste. Although the OTC approach is operationally simple and offers superior proliferation resistance, it still suffers from extremely low resource utilization efficiency. In contrast, the CFC method employs reprocessing to recover and recycle uranium and plutonium, which improves the utilization of fuel and minimizes the volume of radioactive waste.[^7^, ^8^] The CFC method can be categorized as dry reprocessing technologies and wet reprocessing technologies.[^9^, ^10^] Among them, dry reprocessing (e.g., electrolytic refining) is well‐suited for fast reactor metal fuels owing to their enhanced proliferation resistance. However, dry reprocessing is limited to pilot‐scale applications. Alternatively, wet reprocessing represents the mainstream technology for spent fuel reprocessing, relying on its high efficiency and selective solvent extraction.[^11^, ^12^] The PUREX (plutonium uranium redox extraction) process, which is the most mature solvent extraction method, represents the global standard for nuclear fuel reprocessing.[^13^, ^14^, ^15^] While various extraction systems can be employed in the PUREX process, tributyl phosphate (TBP)/alkane diluent system (typically TBP/kerosene or TBP/n‐alkane) is the most widely adopted. The above solvent systems enable effective separation and recovery of uranium/plutonium from nitric acid solutions in industrial‐scale operations while satisfying stringent radioactive material handling and non‐proliferation requirements.[^4^, ^16^, ^17^, ^18^] However, under extreme conditions (e.g., intense radiation, high acidity, and elevated temperature), the solvent inevitably degrades, inducing the generation of undesired byproducts. These degradation products impair extraction efficiency, and their presence may cause equipment corrosion and present challenges to radioactive waste management.[^19^, ^20^, ^21^]

Research into the solvent degradation mechanism and relevant products was initiated in the 1950s. Subsequently, extensive investigation had been conducted to elucidate the degradation behavior of the TBP/diluent system.^[22]^ Recent advances in reprocessing technologies and evolving nuclear fuel cycle requirements have further stimulated extensive studies on spent fuel reprocessing. In this paper, the degradation mechanism of TBP solvent systems during nuclear fuel reprocessing, the product characteristics of the degradation products, and the effect of products on the extraction process are systematically reviewed. Through critically analyzing the characterization technologies (e.g., chromatography, and spectroscopy), key technical challenges are identified, and future research directions are proposed to facilitate process optimization.

## 2 OVERVIEW OF THE PUREX SOLVENT SYSTEM

The PUREX process represents the industrial standard for nuclear fuel reprocessing and is currently widely used for the reprocessing of spent fuel. The core feature of the PUREX process is the utilization of solvent extraction to achieve high‐efficiency separation of uranium, plutonium, and other fission products.[^23^, ^24^] This extraction process utilizes an organic phase containing TBP and a diluent (e.g., kerosene or n‐dodecane) as the extractant, and selective recovery of uranium and plutonium is achieved through a multistage countercurrent extraction process with a back‐extraction step.^[25]^ Notably, the physicochemical properties of the solvent system and the choice of diluent exert a decisive influence on the extraction efficiency, phase separation performance, and process stability. Thus, a systematic understanding of their roles and composition optimization are required.

**FIGURE 2:**
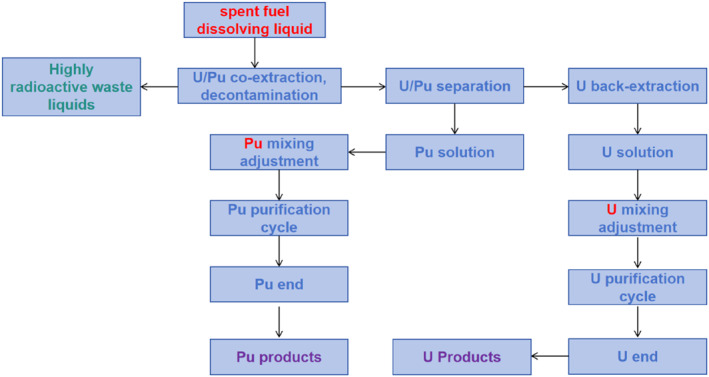
Schematic diagram of the plutonium uranium redox extraction process.^[29]^

The TBP/kerosene system serves as a classic example of a solvent system utilized for fuel reprocessing.[^26^, ^27^, ^28^] TBP is the core extractant in the PUREX process because its phosphate group coordinates with ${\text{UO}}_{2}^{2+}$ and Pu^4+^ ions to form neutral complexes, enabling selective extraction. In general, about 20–30 vol.% TBP is dissolved in the diluent, ensuring sufficient extraction capacity while alleviating phase separation difficulties caused by excessive viscosity. Initially, in a 3–4 M HNO_3_ medium, uranyl (${\text{UO}}_{2}^{2+}$) and plutonium (Pu^4+^) ions are selectively co‐extracted by the TBP/kerosene organic phase, achieving partition coefficients up to 10 and 3, respectively. This separates ${\text{UO}}_{2}^{2+}$ and Pu^4+^ ions from most fission products. Subsequently, a preferential reductive back‐extraction step is employed to reduce Pu^4+^ ions to Pu^3+^ (e.g., using a U(V)‐hydrazine system), which lowers the partition coefficient of Pu to 0.001 and enables plutonium recovery. Finally, uranium is back‐backextracted using dilute HNO_3_ (∼0.01 M). Overall, this multistage countercurrent extraction purification process results in the formation of uranium and plutonium products with purity exceeding 99.9%, meeting the industrial recovery targets of >99.8% for uranium and >99.5% for plutonium. A high‐level waste solution is also generated, requiring vitrification for final disposal (Figure 2).

Owing to the high selectivity, adopting the TBP/kerosene system significantly enhances the distribution ratio of uranium and plutonium over fission products. As a result, the TBP/kerosene system has been the most widely used for spent fuel processing. In addition, this system exhibits excellent chemical stability under strong acid and radiation conditions as well as moderate density and interfacial tension. Simultaneously, the TBP/kerosene system provides highly efficient two‐phase partitioning performance. However, during long‐term operation, TBP may be hydrolyzed to generate degradation products, representing a critical issue that needs to be addressed. One such degradation product is dibutyl phosphate (HDBP),^[30]^ which can easily combine with zirconium, niobium, and other fission elements to form stubborn precipitates that are difficult to dispose of. To meet this challenge, not only processing conditions and should be optimized but also new stabilizers and extraction systems should be developed.

It should be noted that the selection of the diluent in TBP/diluent system is of equal importance. Since the diluent plays multiple roles in addition to adjusting the viscosity, density, and other physical properties of the organic phase, it must remain chemically inert to prevent side reactions. For instance, n‐dodecane is often chosen as a diluent because of its saturated alkane structure, which provides excellent radiation stability. Kerosene is also a popular diluent due to its moderate aromatic content, offering superior solubility to inhibit third‐phase formation.^[31]^ Aiming at practical applications, the properties of diluents need to be evaluated thoroughly. For instance, diluents with high flash point and low volatility, such as hydrogenated kerosene (HOK), can markedly reduce operational risks, athough the economic feasibility of industrial‐scale supply must also be considered. The carbon chain length distribution of diluents also influences their mass transfer efficiency. Specifically, shorter‐chain alkanes generally enhance the extraction kinetics, but their high volatility may lead to volatile losses. Balancing these complex factors is crucial for achieving an efficient and stable extraction process.

To summarize, the TBP/kerosene system has become the standard solvent configuration in the PUREX process, owing to its balanced extraction performance and operational stability. However, the long‐term durability of the TBP/diluent system remains a grand challenge. In the future, research should be focused on exploring novel extractants or diluents capable of providing enhanced irradiation resistance; simultaneously, TBP alternatives should be developed to minimize the accumulation of degradation products. Solvent systems must synergistically optimize chemical efficiency, engineering feasibility, and economic cost to support the sustainability of the nuclear fuel cycle.

## 3 DEGRADATION OF PRODUCTS AND MECHANISMS OF TBP/DILUENT SYSTEMS

In the PUREX process, TBP/diluent systems primarily comprise two phases. The organic phase comprises 20%–30% TBP in an inert diluent such as kerosene or n‐dodecane, while the aqueous phase is composed of aqueous HNO_3_ solution (3–4 M). Thus, degradation products mainly originate from the radiolysis of TBP and diluent.

### 3.1 TBP irradiation: Degradation products and mechanisms

As a widely adopted extractant in nuclear fuel reprocessing, TBP exhibits both chemical and radiological stability, enhancing the operational safety and efficiency of the PUREX process. However, during the PUREX process, TBP is subjected to intense radiation and aggressive chemical conditions, which can lead to its chemical and radiolytic degradation, inevitably resulting in performance deterioration. Other reported degradation mechanisms include oxidation, nitrolysis, and pyrolysis.^[32]^ Nevertheless, radiolysis predominates under the intense radiation conditions of the nuclear fuel cycle. Therefore, process efficiency is negatively affected by the radiolysis products of TBP.

**FIGURE 3:**
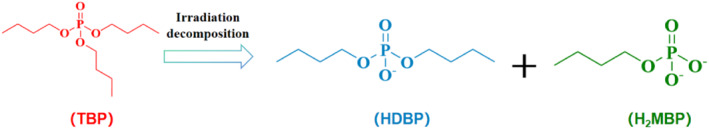
Chemical structures of TBP and its degradation products HDBP and H_2_MBP.^[33]^ HDBP, dibutyl phosphate; TBP, tributyl phosphate.

Descriptive review of individual studies: An experimental study by Mincher et al.^[33]^ demonstrated that irradiating 30% TBP in an alkane diluent with HNO_3_ solution induced the generation of diverse radiolysis products, including HDBP, monobutyl phosphate (H2MBP), phosphoric acid, high‐molecular‐weight dimers, acid phosphates, hydroxyphosphates, and nitro‐substituted phosphates (Figure 3). Among them, the high‐molecular‐weight products predominantly formed via radical addition reactions, while HDBP and the low‐molecular‐weight acid phosphates were generated via TBP radical cation radiolysis or reactions involving primary radiolysis products abstracting hydrogen atoms from intact TBP molecules. In addition, the combination of oxygen molecules with carbon‐centered radicals resulted in the formation of peroxyl radicals, which initiated chain oxidation reactions. Similarly, nitrogen‐centered radicals may react with carbon‐centered radicals to yield nitration products. Although the radiation source type and dose rate both influence product yields, they do not alter the fundamental chemical identity of the radiolysis products.

**FIGURE 4:**
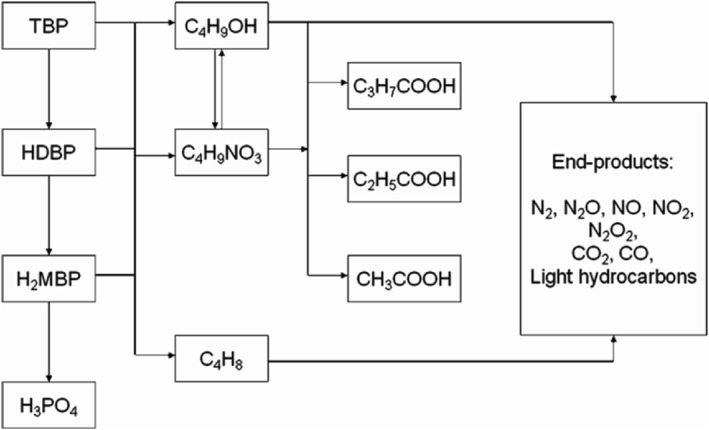
Simplified schematic diagram of TBP degradation products.^[32]^ TBP, tributyl phosphate.

Tahraoui and Morris^[34]^ had reviewed on the degradation behavior of TBP/diluent systems focusing on the chemical and radiation degradation mechanisms of TBP and its diluents. They reported that hydrolysis, which was the main degradation pathway of TBP, generated products such as HDBP and butanol. Among them, HDBP could be further hydrolyzed, while butanol may undergo a nitrification reaction to generate butyl nitrate or an oxidation reaction, leading to its eventual conversion into carboxylic acids. It was noted that all of these degradation products may undergo pyrolysis at elevated temperature, ultimately leading to the generation of gaseous end products as listed in Figure 4.

Zhang^[35]^ pioneered the use of an α‐irradiation platform to investigate the radiolysis behavior of TBP. It was demonstrated that the yields of H_3_PO_4_, H_2_MBP, and HDBP increased with increasing radiation dose (0–400 kGy) and HNO_3_ concentration (0.01–5 M) with 1 M HNO_3_ concentration sufficient to achieve H_3_PO_4_ and H_2_MBP saturation. This study revealed that both water and Zr (IV) inhibited the radiolytic degradation of TBP, while HOK showed a similar suppression effect at doses exceeding 100 kGy. Sarkar et al.^[36]^ employed Fourier transform infrared (FT‐IR) spectroscopy and gas chromatography‐mass spectrometry (GC‐MS) to characterize trialkyl phosphate degradation under γ‐irradiation. Their FT‐IR analysis identified various radiolysis products, including alkyl nitrates, carboxylic acids, and nitroalkanes. Meanwhile, GC‐MS analysis was employed to detect the diluent degradation products (e.g., dodecanol and nitro‐dodecane) and characteristic compounds such as dialkyl phosphates. Lloyd and Fellows^[37]^ investigated the influence of α‐on a 30% TBP/nor‐dodecane system at different temperatures, revealing that the temperature significantly affected both the chemical hydrolysis and radiolysis rates of TBP. The above data indicated that HDBP was generated as the predominant product below 50°C, while H_2_MBP generation rate markedly increased at 80°C. This suggested that H2MBP formation via TBP radiolysis was enhanced at elevated temperatures. In addition, Pu's dual promotion mechanism (direct α‐irradiation effects and metal ion‐catalyzed hydrolysis) for TBP radiolysis was elucidated.

The type of irradiation has been reported to significantly affect the radiolysis of the TBP extraction system. At present, most studies on the radiolytic stability of TBP have focused on low‐LET (linear energy transfer) irradiation and electron accelerator experiments. In contrast, the radiolytic behavior of TBP under high‐LET conditions has rarely been explored. Pearson et al.^[38]^ performed comparative experiments under low‐LET (γ‐radiation) and high‐LET (simulated α‐radiation) conditions. Under low‐LET conditions, both the degradation rate of TBP (0.36 μmol/J) and the HDBP yield (0.18 μmol/J) were significantly higher than the values under high‐LET conditions (0.14 and 0.047 μmol/J, respectively), confirming that the damaging effect of α‐radiation on the TBP system was relatively weak compared with that of γ‐radiation.

n addition to studies on TBP degradation products, the relevant mechanism of degradation was evaluated. TBP is degraded under radiation conditions, where molecular bonds are broken by the radiation energy. This process generates free radicals and fragmentation products, while free radicals further react to form small molecules such as butanol, HDBP and H_2_MBP. Simultaneously, hydrogen, methane, and other gases may be released. This degradation process gradually destroys the chemical structure of TBP, which negatively affects the extraction performance, and may lead to the generation of acidic products. The studies discussed in this section evaluate the complex mechanisms of TBP radiation degradation.

Relevant studies on the radiation‐induced degradation of organophosphates proposed mechanisms involving ionic and radical reactions. For instance, Wilkinson and Williams^[39]^ found that irradiating TBP with electrons mainly produced HDBP with absolute yields decreasing as the alkyl chain length increased. H_2_MBP production showed a tenfold decrease under electron irradiation, while only negligible amounts of phosphoric acid were formed. The yields of hydrocarbon byproducts (olefins and alkanes) did not match the organic fragments lost during acid formation, while the hydrogen yields typically increased with increasing ester molecular weight. MS analysis revealed that the organophosphate ions underwent rearrangement via alkyl chain scission, representing a process that competed with acid formation through alternative liquid‐phase ionic‐pathways. They proposed an ionic mechanism involving six or seven‐membered ring transition states that decomposed into protonated dialkyl phosphates and olefinic radicals, ultimately yielding dialkyl phosphate salts. The above study established a fundamental mechanistic framework for understanding phosphate solvent radiolysis in nuclear fuel reprocessing. Haase et al.^[40]^ utilized electron spin resonance (ESR) tests to verify that organic phosphate esters mainly produced two types of radicals under low‐temperature radiation: Alkyl radicals were directly generated by the reaction of an electron with the C‐O bond, while positively charged molecules were formed via reactions with hydrogen atom radicals. Notably, their work demonstrated that TBP underwent preferential γ‐site hydrogen abstraction. These findings provide insights into the decomposition of organophosphate esters under radiation. Subsequent studies[^41^, ^42^] further expanded the radical reaction mechanism by confirming that electron capture enabled production of both butyl radicals and HDBP. Kuruc et al.^[43]^ employed ESR to demonstrate the existence of peroxyl radicals. Their findings revealed that X‐ray irradiation triggered the fracture of the TBP molecules inducing the generation of two types of radicals, that is, alkyl radicals (e.g., CH_3_ĊHCH_2_‐) and peroxy radicals generated by a reaction with oxygen. Moreover, X‐rays generated quantified radical yields (12.9 radicals/100 eV) than γ‐rays (6.8 radicals/100 eV). Deuterium labeling studies confirmed that butyl radicals were formed in a process involving γ‐site intramolecular hydrogen transfer. Simultaneously, the HNO_3_ environment in the PUREX process triggered a nitration reaction and the chain degradation of TBP, leading to the generation of nitrophosphate that significantly affected the extraction performance. In summary, the above studies elucidated the complex degradation behavior of organophosphates under radiation at the molecular level.

It should be noted that more recent discovery of free radical pathways and the subsequent study of PUREX process system provided key scientific basis for the screening and process optimization of extractants in nuclear fuel reprocessing. In particular, the discovery of γ‐site hydrogen capture and nitration reactions directly guided the molecular design and process control of radiation‐resistant extractants.

Systematic comparative analysis of degradation behavior: A critical synthesis of the existing literature requires moving beyond a sequential recounting of individual studies. Understanding TBP degradation necessitates a systematic analysis that examines how key variables—radiation type, chemical environment (particularly acidity), and temperature—interact to determine both the extent of degradation and the resulting product distribution. To enable meaningful cross‐study comparisons, the radiation chemical yield, or *G*‐value (typically expressed in μmol/J), serves as the fundamental quantitative metric. It represents the amount of a substance produced or destroyed per unit of absorbed energy. Table 1 consolidates these essential parameters, providing the data framework for the comparative analysis that follows.

**TABLE 1:** Summary of key parameters and mechanisms for TBP degradation under different irradiation conditions.

Influencing factor	Experimental conditions	TBP degradation *G*‐value (μmol/J)	Main product yields (*G*‐value, μmol/J)	Key conclusions and mechanistic insights	References
Radiation type (LET)	γ‐radiation (low LET)	0.36	HDBP: 0.18	Conclusion: Low‐LET radiation produces sparsely distributed, highly reactive radicals, leading to efficient chain degradation and higher apparent degradation efficiency	Pearson et al.^[38]^
	α‐radiation (high LET)	0.14	HDBP: 0.047	Conclusion: High‐LET radiation generates extremely high local radical concentrations, increasing radical‐radical recombination probability and reducing apparent degradation efficiency. More closely mimics the actual plutonium‐bearing radiation field	Pearson et al.^[38]^
Acidity [HNO_3_]	Low to medium conc. (0.01–5 M)	Increases with acidity	Yields of H_3_PO_4_, H_2_MBP increase with acidity, saturating at ∼1 M	Conclusion: H^+^ significantly promotes the formation of deep degradation products (H_3_PO_4_, H_2_MBP). Saturation suggests equilibrium in hydrolysis or depletion of active intermediates	Zhang^[35]^
	High conc. (>3 M)	Relatively high yields	HDBP, H_2_MBP, nitro‐phosphates, etc.	Conclusion: High‐acidity environments strongly promote hydrolysis and nitration reactions, generating a more complex product spectrum	Mincher et al.^[33]^
Temperature	<50°C	Rate influenced by temperature	HDBP as the main product	Conclusion: Radiolysis is the dominant mechanism, producing primary degradation products like HDBP	Lloyd and Fellows^[37]^
	>80°C	Rate influenced by temperature	H_2_MBP yield increases significantly	Conclusion: Chemical hydrolysis is markedly activated, promoting deep dealkylation (HDBP → H_2_MBP), indicating a significant thermo‐radiolytic synergistic effect	Lloyd and Fellows^[37]^
Other factors	Presence of water & HNO_3_	Not explicitly reported	HDBP, H_2_MBP, H_3_PO_4_, carboxylic acids, nitroalkanes, etc.	Conclusion: Water provides hydrolyzing agents and active species like ·OH, leading to a more complex product spectrum. HNO_3_ introduces nitration pathways	Mincher et al.,^[33]^ Tahraoui and Morris^[34]^
	Presence of Zr(IV)	Degradation is suppressed	Yields of HDBP, H_2_MBP are reduced	Conclusion: Zr(IV) may inhibit TBP radiolysis, possibly via complexation or radical scavenging	Zhang^[35]^
	Pure TBP (electron irradiation)	Not explicitly reported	HDBP as main product; H_2_MBP yield an order of magnitude lower	Conclusion: Under anhydrous conditions, an ionic mechanism (via six‐ or seven‐membered ring transition states) is an important pathway for HDBP formation	Wilkinson and Williams^[39]^

*Note*: *G*‐value: Radiation chemical yield, defined as the amount of substance produced or destroyed per unit of absorbed energy (unit: μmol/J). It is the key parameter for comparing radiolytic efficiency. The *G*‐values listed are typical ranges; specific values can vary with dose rate, diluent, atmosphere, etc.

Abbreviations: α‐particles, high‐LET radiation; γ‐photons, low‐LET radiation; HDBP, dibutyl phosphate; LET, linear energy transfer; TBP, tributyl phosphate.

The comparative analysis in Table 1 elucidates several fundamental patterns and trade‐offs. First, regarding radiation type, the data reveal a clear efficiency‐selectivity trade‐off. γ‐irradiation, with its higher *G*‐values, is an efficient tool for laboratory‐scale mechanistic studies and accelerated aging tests. In contrast, α‐irradiation, while exhibiting lower degradation yields, provides a more realistic simulation of the high‐LET radiation field present in real spent fuel reprocessing, making its data crucial for long‐term performance prediction. Second, the role of the chemical environment is paramount. Acidity acts not merely as a corrodent but as a catalyst for degradation pathway branching. The promotion of deep hydrolysis products (H_2_MBP, H_3_PO_4_) at moderate acidity and the initiation of nitration pathways at high acidity demonstrate how the chemical medium directs the degradation fate. Third, temperature emerges as a critical switch governing mechanistic dominance. The shift from a radiolysis‐dominated regime at lower temperatures (producing HDBP) to a hydrolysis‐accelerated regime at elevated temperatures (favoring H_2_MBP) underscores a temperature‐induced pathway switching. This explains the severe solvent degradation observed in process hot spots like evaporators. Finally, the presence of other species, such as Zr(IV), introduces inhibitory effects, highlighting that the system's complexity necessitates considering both promoting and inhibiting interactions. This systematic comparison thus transcends individual observations, providing a unified framework to understand, predict, and mitigate TBP degradation by identifying the levers that most significantly control its rate and direction.

### 3.2 Diluents irradiation: Degradation products and mechanisms

In TBP/diluent extraction systems, diluents were mainly employed to regulate the physicochemical properties of the organic phase (e.g., viscosity, density, polarity, and extraction selectivity). The choice of diluent directly affected the extraction efficiency, phase separation behavior, and irradiation resistance of the TBP/diluent system. Inert hydrocarbons such as kerosene, n‐dodecane, and toluene were commonly employed as diluents.^[44]^ Alkane diluents typically exhibit excellent chemical stability, while irradiation may induce the generation of free radicals. In contrast, aromatic diluents would enhance the solubility of TBP at the expense of being more susceptible to degradation.^[45]^ Therefore, mixed diluents (e.g., kerosene‐dodecane) are commonly used to balance viscosity, polarity, and irradiation stability.

**FIGURE 5:**
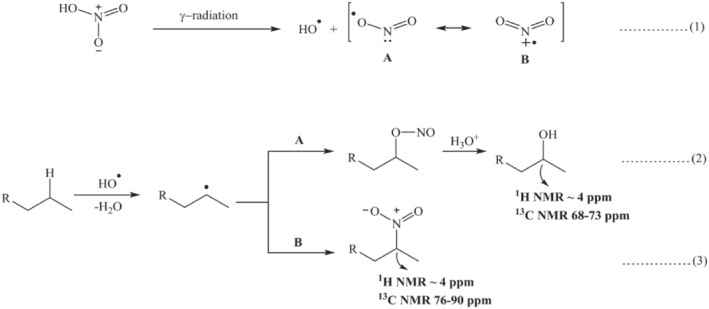
Reasonable reaction mechanism of the formation of dodecyl alcohol and nitro‐dodecane.^[47]^

Mincher et al.^[33]^ reported that the irradiation of diluents would lead to their conversion into alkane adducts, nitroalkane derivatives, and oxidation products (e.g., ketones and carboxylic compounds). Tripathi and Ramanujam^[46]^ further demonstrated that the density and viscosity of 30% TBP‐n‐dodecane‐HNO_3_ mixtures significantly increased with increasing HNO_3_ concentration and radiation dose. This could be attributed to radiation‐induced diluent polymerization, nitration reactions, and hydrogen bonding. Mishra et al.^[47]^ conducted γ‐irradiation experiments on a 30% TBP/n‐dodecane system saturated with 4 M HNO_3_ to replicate standard PUREX process conditions (Figure 5). The presence of HNO_3_, water and dissolved oxygen in this irradiated reaction system may produce a variety of oxidation products. This degradation reaction mainly generated two mixtures: One was a mixture of three dodecanols, namely dodec‐3‐ol, dodec‐4‐ol, and dodec‐5‐ol in a ratio of 2:1:6. The other was a mixture of four nitro‐dodecanes, 2‐nitrododecane, 3‐nitrododecane, 4‐nitrododecane, and 5‐nitrododecane in the ratio of 2:2:1:1. Sequential alkaline washing, vacuum distillation, and column chromatography were employed to isolate and characterize the above products, demonstrating their substantial impact on solvent performance. Notably, the zirconium retention values reached 1740 ppm. This work provided dual insights: On the one hand, the molecular‐level degradation pathways in PUREX solvents were elucidated; on the other hand, the crucial role of these pathways in the deterioration of solvent performance was highlighted. These findings established a fundamental understanding of PUREX solvent degradation while offering theoretical guidance for post‐treatment process optimization.

Stieglitz and Becker^[48]^ identified alkanes in the presence of HNO_3_, confirming the formation of nitroalkanes and nitrosoalkanes (RNO_2_ and RONO_2_). In addition, Cong et al.^[49]^ investigated the α‐irradiation of TBP in different aliphatic diluents and found that kerosene better protected TBP than n‐dodecane and HOK. However, with increasing absorbed dose (up to 200 kGy), this protective effect completely disappeared. The above findings showed that the chemical stability and irradiation behavior of diluents decisively influenced the long‐term performance of the TBP system.

## 4 CHARACTERIZATION TECHNIQUES FOR EVALUATING DEGRADATION PRODUCTS

The comprehensive characterization of TBP degradation products, including small organic molecules (butanol, olefins), phosphate derivatives (HDBP, H_2_MBP), and inorganic species (${\text{PO}}_{4}^{3-}$), is fundamental for elucidating degradation mechanisms and assessing the performance of the TBP/diluent system. Chromatography, spectroscopy, MS, and computational chemistry‐assisted resolution were commonly employed to analyze relevant complex products.

Spectroscopic techniques[^50^, ^51^] are crucial for characterizing the degradation products of TBP. For instance, FT‐IR spectroscopy (FT‐IR) could be employed to quickly identify the changes in the hydroxyl groups, phosphate ester groups, and other functional groups of TBP degradation products. Thus, FT‐IR analysis provided an intuitive understanding of the chemical bonds that broke during the degradation process. FT‐IR analysis was complemented by Raman spectroscopy, which was sensitive to the symmetric vibration of molecules while did not experience interference from water molecules. Thus, Raman spectroscopy was suitable for monitoring the changes of phosphate ester bonds in the aqueous phase system. Meanwhile, nuclear magnetic resonance (NMR) spectroscopy could provide information on the structural environment at the atomic level, enabling accurate identification and quantification of derivatives such as HDBP and H_2_MBP.

Chromatographic techniques also played a decisive role in characterizing TBP degradation products. By combining GC‐MS, high‐performance liquid chromatography (HPLC) and ion chromatography (IC), systematic detection of volatile organic compounds, polar derivatives, and inorganic ions could be realized.[^52^, ^53^] GC‐MS was mainly used to accurately identify volatile degradation products such as C_4_–C_8_ alkanes, butenes, and butanols. Performing HPLC with a reversed‐phase C_18_ column enabled the separation and detection of polar products, such as HDBP, H2MBP, and organic acids. IC was utilized for accurate identification of inorganic ions such as ${\text{PO}}_{4}^{3-}$ and ${\text{NO}}_{3}^{-}$.

The use of high‐resolution mass spectrometry (HRMS) techniques significantly increased the depth and breadth of degradation product analysis.[^54^, ^55^] While conventional MS utilized characteristic ion peaks to achieve initial product degradation, the mass accuracy of HRMS could be <5 ppm. HRMS enabled accurate determination of molecular formula, which was quite effective in distinguishing isomers. The coupling and cross‐validation of multiple detection methods significantly supported an in‐depth understanding of the TBP degradation mechanism and process optimization.

**FIGURE 6:**
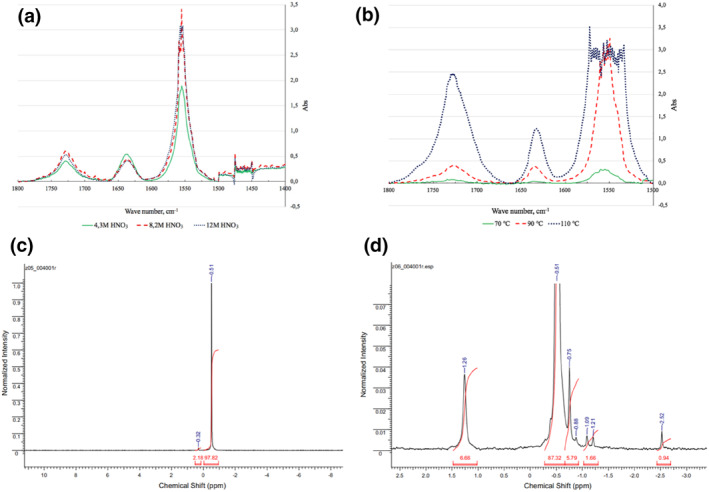
(a) FT‐IR spectra of 4.3, 8.2 and 12.0 M HNO_3_ systems; (b) FT‐IR spectra of 8.2 M HNO_3_ system at temperatures of 70°C, 90°C, and 110°C; (c) NMR spectrum of the organic phase after pyrolysis of 8.2 M HNO_3_ system at 70°C, and a high pressure; (d) NMR spectrum of the organic phase of 8.2 M HNO_3_ system after pyrolysis at 110°C and a high pressure.^[61]^ FT‐IR, Fourier transform infrared; NMR, nuclear magnetic resonance.

Levitskaia et al.^[56]^ employed FT‐IR to differentiate between HDBP and TBP in nitric acid solvents containing TBP/n‐dodecane. Kuzmin et al.^[57]^ employed NMR, infrared spectroscopy, and GC‐MS analyses to confirm that the decomposition of TBP mainly produced butene and a complex mixture of alkanes/olefins (containing C_8_–C_16_ fractions). The butene yields in their study varied from 2% to 50% as the process conditions changed. Hardy and Scargill^[58]^ utilized FT‐R spectroscopic analysis to reveal the bidentate coordination feature of ${\text{NO}}_{3}^{-}$, which was certificated by the presence of characteristic peaks at 1557 and 1272 cm^−1^. Meanwhile, peak migration from 1228 to 1120 cm^−1^ indicated the interaction of P=O bonds with Zr, while the disappearance of characteristic peaks of hydrogen bonds proved that deprotonated HDBP participated in the coordination structure. Tashiro et al.^[59]^ employed GC, IC, and GC‐MS to quantify the degradation products in organic aqueous phase of a TBP/diluent system, identifying non‐phosphate ester products such as butyl nitrate, butanol, and diverse carboxylic acids. Concentration variation of key intermediate HDBP was determined by inductively coupled plasma spectroscopy. Lesage et al.^[60]^ used tandem mass spectrometry (MS‐MS) and gas chromatography‐tandem mass spectrometry (GC‐MS/MS) to convince that under oxidative conditions in the presence of HNO_3_, TBP radicals were dimerized and then converted into hydroxyl/nitro substituents such as butylphosphate, alkylated TBP, and other derivatives. Among them, the butyl chain hydroxylation products further formed ketones, ethers and O‐esters. Under these conditions, 3‐hydroxy TBP was the main intermediate due to its special stability, while 3‐nitro TBP exhibited excellent stability due to its six‐membered ring configuration. Dzhivanova et al.^[61]^ utilized FT‐IR and NMR to characterize the radiolytic and thermal decomposition products of a 30% TBP‐Isopar (isoparaffin)‐M extraction system in HNO_3_ environments (Figure 6). According to the FT‐IR analysis, the major degradation products in this system included nitro compounds, organic nitrates, carboxylic acids, ketones, and esters with nitro compounds and carboxylic acids predominating. ^31^P NMR analysis showed that the degradation products of TBP were mainly HDBP and H2MBP, while the concentrations of these two compounds increased with increasing radiation dose (0.5–2 MGy) and temperature (70–110°C).

Jala et al.^[62]^ characterized irradiated TBP using FT‐IR, NMR and GC‐MS techniques. FT‐IR analysis showed that the bound water molecules gradually disappeared from TBP as the irradiation dose increased, but the carbon skeleton structure remained intact. The retention of the TBP carbon skeleton structure was confimed by ^13^C NMR and ^1^H NMR analyses. According to the GC‐MS results, high‐dose irradiation induced the generation of irradiation products, such as HDBP and trace hydrocarbon products as well as high‐molecular‐weight polymers. Tripathi et al.^[63]^ used GC, FT‐IR and GC‐MS techniques to characterize the irradiation products of a 30% TBP‐n‐dodecane‐HNO_3_ system. The GC spectrum shoued the characteristic peaks of nitroalkanes, long‐chain alcohols, and nitrate esters, and FT‐IR analysis confirmed the generation of nitrogen‐containing products. Meanwhile, GC‐MS confirmed that C_8_‐C_12_ alcohols and polymerization products were dominant. Tallent et al.^[64]^ analyzed the chemical degradation behavior of an n‐alkane (NPH) diluent in a 30% TBP system by GC, FT‐IR, and GC‐MS. The GC analysis identified nitroalkanes (RNO_2_), nitrites (RONO), and alcohols (ROH) as the main degradation products, while the characteristic peaks of nitro and nitrite groups were detected by FT‐IR at 1565 and 1640 cm^−1^, respectively. They confirmed the presence of nitrogen compounds in the degradation products. Secondary products, such as unsaturated alcohols, nitroalcohols, ketones, and carboxylic acids were also detected by GC‐MS. These findings provide an important experimental basis for studying the degradation mechanisms of nuclear fuel reprocessing solvents.

To facilitate the selection of appropriate analytical methods, a comparative evaluation of the major techniques is provided in Table 2, detailing their respective strengths, limitations, and primary applications for specific degradation products.

**TABLE 2:** Comparative analysis of characterization techniques for TBP and degradation products.

Technique	Target analytes/information	Key strengths	Main limitations	Typical application scenario
FT‐IR	Functional groups (P=O, P–O–C, NO_2_, OH, C=O)	Rapid, non‐destructive. In‐situ capability for reaction monitoring. Sensitive to bond vibration changes	Limited specificity for complex mixtures. Difficult for quantitative analysis of trace components. Interference from water (requires careful sample handling)	Initial screening for bond cleavage (e.g., P=O weakening), identification of nitro/hydroxy/carboxyl groups, and monitoring hydrolysis/nitration progress
NMR (^31^P, ^1^H, ^13^C)	Molecular structure, coordination environment, quantification of HDBP/H_2_MBP, TBP	Definitive structural elucidation. Quantitative without standards (for known compounds). Probes metal‐ligand interactions in solution	Low sensitivity (requires concentrated samples). Expensive and complex operation. Not suitable for trace analysis or highly radioactive samples without specialized equipment	Quantifying HDBP/H_2_MBP ratios, confirming degradation product structures, studying complexation with metal ions (e.g., U, Pu, Zr)
GC‐MS	Volatile and semi‐volatile organics (C4–C16 alkanes/alkenes, butanols, alkyl nitrates, nitroalkanes)	Excellent separation and definitive identification via mass spectra. High sensitivity for trace VOCs. Extensive library support	Requires volatility or derivatization (unsuitable for HDBP/H_2_MBP directly). Thermal decomposition risk for labile compounds. Not ideal for polar, non‐volatile species	Identifying diluent degradation products (dodecanols, nitro‐dodecanes), volatile by‐products (butyl nitrate), and hydrocarbon fragments
HPLC (with various detectors)	Polar, non‐volatile compounds (HDBP, H_2_MBP, carboxylic acids, polar nitro‐products)	Handles thermally labile and polar compounds directly. Good quantitative capability. Couples with MS (LC‐MS) for advanced analysis	Less efficient separation than GC for very complex volatile mixtures. Method development can be time‐consuming	Quantitative analysis of HDBP and H_2_MBP in solvent streams, separation of polar acidic degradation products
IC	Inorganic anions (${\text{PO}}_{4}^{3-}$, ${\text{NO}}_{3}^{-}$, ${\text{NO}}_{2}^{-}$) and organic acids	High selectivity and sensitivity for ions. Direct analysis of aqueous phases. Excellent for quantifying inorganic phosphate	Limited to ionic/polar species. Requires aqueous or appropriately dissolved samples	Monitoring final hydrolysis products (${\text{PO}}_{4}^{3-}$), quantifying nitrate/nitrite from nitration, analyzing aqueous waste streams
HRMS (LC‐HRMS, GC‐HRMS)	Molecular formula, unknown degradation products, complex adducts, isomers	Unmatched mass accuracy for formula assignment. Powerful for identifying unknowns and distinguishing isomers. Essential for elucidating novel degradation pathways	Very high cost and operational complexity. Requires expert interpretation. Often used as a complementary, not routine, technique	Discovery and identification of novel/non‐routine degradation products, confirming structures of complex metal‐ligand species (e.g., with ESI‐MS)

Abbreviations: ESI‐MS, electrospray ionization mass spectrometry; FT‐IR, Fourier transform infrared; GC‐MS, gas chromatography‐mass spectrometry; HDBP, dibutyl phosphate; HPLC, high‐performance liquid chromatography; HRMS, high‐resolution mass spectrometry; IC, ion chromatography; NMR, nuclear magnetic resonance; TBP, tributyl phosphate.

## 5 COMPLEXATION OF DEGRADATION PRODUCTS AND THIRD‐PHASE FORMATION

### 5.1 Complexation of degradation products with metal ions

**FIGURE 7:**
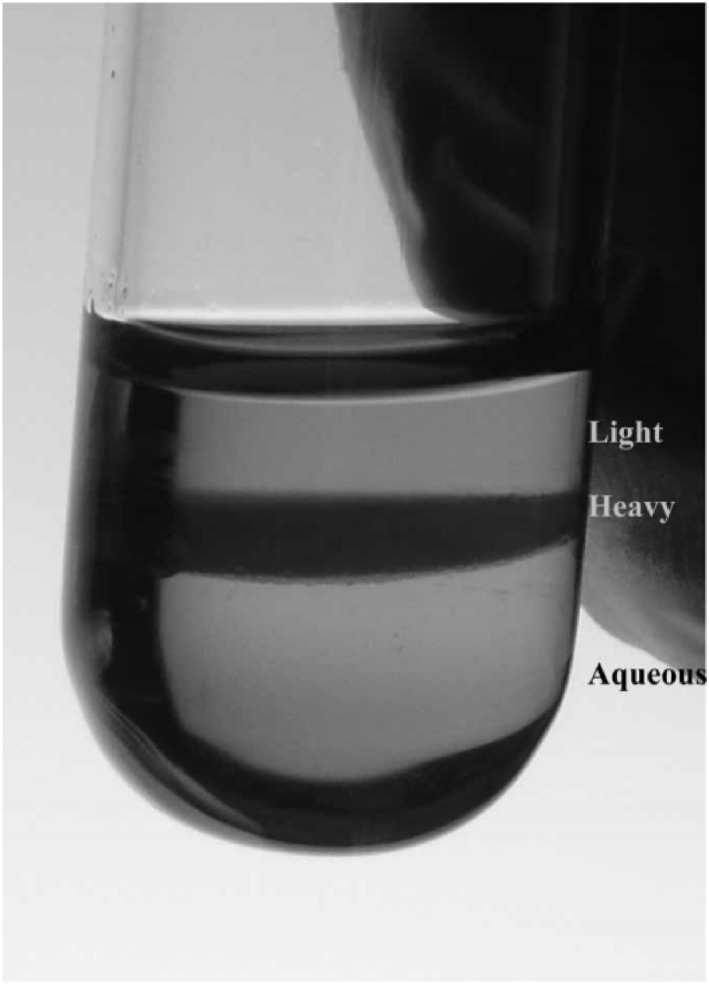
Formation of a third phase in 1.1 M, TBP/HPT solution.^[65]^ HPT, hydro‐genated polypropylene tetrame; TBP, tributyl phosphate.

It is well known that under specific solvent extraction conditions, density differences within the organic phase can lead to the formation of two liquid phases: a heavy phase rich in extractant, acid, and metals, and a light phase comprised mainly of diluent (Figure 7). In the PUREX process, the HDBP and H_2_MBP byproducts generated by TBP degradation would interact with target metal ions such as uranium (U) and plutonium (Pu), significantly affecting their extraction efficiency. HDBP and H_2_MBP would strongly coordinate with these metal ions with HDBP notably forming the stable neutral complex Pu(DBP)_4_ with tetravalent plutonium (Pu^4+^). These complexes exhibited low solubility in the organic phase and tended to remain in the extraction system, which negatively affected plutonium recovery.^[66]^ In addition, HDBP and H_2_MBP also competitively coordinated with uranyl ions (${\text{UO}}_{2}^{2+}$) to form complexes such as UO_2_(DBP)_2_ or UO_2_(MBP)_2_, which effectively reduced the uranium extraction capacity of TBP for uranium.^[67]^ The presence of these phosphate degradation products lowered the metal loading capacity of the organic phase, which in turn affected the separation and purification of uranium/plutonium.

Gao et al.^[68]^ studied the degradation of a TBP/n‐dodecane system under α‐radiation, concluding that the radiation‐generated degradation products, such as HDBP and H_2_MBP, formed stable complexes with Pu^4+^ and reduced the effective concentration of TBP. Further increasing the radiation dose led to a significant increase in Pu retention (e.g., a radiation dose of 1 × 106 Gy state led to 37.5% Pu retention). Metal ion competition experiments showed that the complexation abilities of the metal ions followed the order Zr^4+^ > ${\text{UO}}_{2}^{2+}$ > Pu^4+^. It was found that Zr^4+^ both promoted TBP degradation and reduced Pu residues via strong complexation, while ${\text{UO}}_{2}^{2+}$ preferentially bound to the degradation products to promote Pu back‐extraction. Moreover, 1–5 M HNO_3_ solutions did not significantly affect the complexation behavior of this system. In another study on the stability of a TBP/kerosene/3 M HNO_3_ system under α (plutonium) and γ (^60^Co) radiation, Gao et al.^[69]^ reported that it was difficult to assess the effect of removal of degradation products via plutonium retention. Plutonium retention tended to monotonically increase with increasing α/γ absorbed dose and was more pronounced in samples with low TBP concentrations, suggesting that the kerosene degradation products formed stronger complexes with plutonium. Upon introducing ${\text{UO}}_{2}^{2+}$ and Zr^4+^, plutonium retention did not significantly change with changing metal ion concentration. However, a comparative experiment revealed that at the same dose, retention followed the order Pu^4+^ samples < ${\text{UO}}_{2}^{2+}$ samples < Zr^4+^ samples. This suggested that ${\text{UO}}_{2}^{2+}$ and Pu^4+^ may compete with each other to form complexes with the degradation products, while Zr^4+^ may promote the irradiation of TBP. Ngelale et al.^[70]^ found that HDBP, which was the main TBP degradation product after exposure to γ or α‐radiation, bound to fission products and affected the extraction process. The presence of uranyl ions accelerated the radiolysis of TBP complexes, but the effect of HNO_3_ on the degradation of these complexes was less significant. These degradation products may form interfacial precipitates, leading to a decrease in the efficiency of nuclide separation efficiency. McDonald et al.^[71]^ utilized electrospray ionization mass spectrometry to perform an in‐depth analysis of the interactions of uranium and plutonium with TBP and its degradation product DBP in the PUREX process with a focus on molecular mechanism of the third‐phase formation. They found that DBP could form stable complexes with high coordination numbers (e.g., [UO_2_(DBP)_3_]^−^ and [PuO_2_(HDBP)_4_]^+^) with uranium/plutonium.

In addition to forming coordinated complexes with the target metal ions, TBP degradation products could also form stable complex third‐phase interfaces with fission‐generated metal ions such as Zr, Nb and Ru. The formation of these third‐phase interfaces negatively affected the mass transfer efficiency and the phase separation process. Addressing third‐phase formation increased the complexity of process control, which had multiple impacts on extraction and separation.^[72]^ George et al.^[73]^ demonstrated that HDBP and Zr^4+^ (2:1 M ratio) formed hydrophobic Zr(NO_3_)_2_(HDBP)_2_(OH)_2_ precipitates that preferentially accumulated in the organic phase and at interfaces, while Zr(HMBP)_2_(OH)_2_ complexes generated from H_2_MBP exhibited higher tendency to precipitate. Blazheva and Zilberman^[74]^ elucidated the Zr extraction mechanism in the HDBP‐TBP‐HNO_3_ system, identifying that Zr overloading led to 1:1 Zr‐HDBP covalent/coordinate bonding, which produced mixed complexes such as Zr(NO_3_)_2_(DBP)_2_. Frydrych et al.^[75]^ revealed a dual mechanism by which the TBP degradation product HDBP triggered the formation of a third phase in the PUREX process. On the one hand, HDBP formed an insoluble complex with Zr(IV)/Pu(IV) to directly form a precipitate such as Zr(DBP)_2_(NO_3_)_2_. On the other hand, TBP underwent intramolecular degradation under thermal/radiation conditions, releasing the explosive compound butyl nitrate (BuONO_2_) and producing metal‐dialkyl phosphate polymers. Kumar and Koganti^[76]^ reported the formation mechanism of the actinide third phase in the TBP system. Relevant spectroscopic analysis showed that base solvates (UO_2_(NO_3_)_2_·2TBP) reacted with HNO_3_‐TBP to form extended solvates (UO_2_(NO_3_)_2_·2TBP·HNO_3_). Simultaneously, the relevant study in this section revealed the complex interaction mechanism of TBP degradation products with metal ions as well as their effects on the extraction process.

### 5.2 Strategies for mitigating effects of third‐phase formation

The formation of the third phase in the TBP system was influenced by multiple factors such as acidity, metal ions, and the nature of diluent. Different strategies have been developed to mitigate the negative effect of third‐phase formation on the TBP system.

One strategy involved the design of improved diluents to suppress the formation of a third phase. For example, Plaue et al.^[65]^ investigated the formation mechanism of a Pu third phase in a 30% TBP/HNO_3_/hydro‐genated polypropylene tetrame (HPT) system. They found that the presence of Pu(VI) in the aqueous phase of 7 M HNO_3_ significantly reduced the metal concentration required to initiate phase splitting in the organic phase. Pu(VI), which existed as plutonium acyl trinitrate (${\text{PuO}}_{2}{\left({\text{NO}}_{3}\right)}_{3^{-}}$), formed a special complex with TBP (confirmed by a characteristic peak at 820 nm). However, despite the enhanced anti‐phase splitting properties of HPT, the utilization of this diluent may reduce the radiation stability compared with dodecane. Su et al.^[77]^ reported a new hyperbranched alkane diluent (HAD), and relevant experiments showed that HAD inhibited third‐phase formation more strongly than n‐dodecane and kerosene.

**FIGURE 8:**
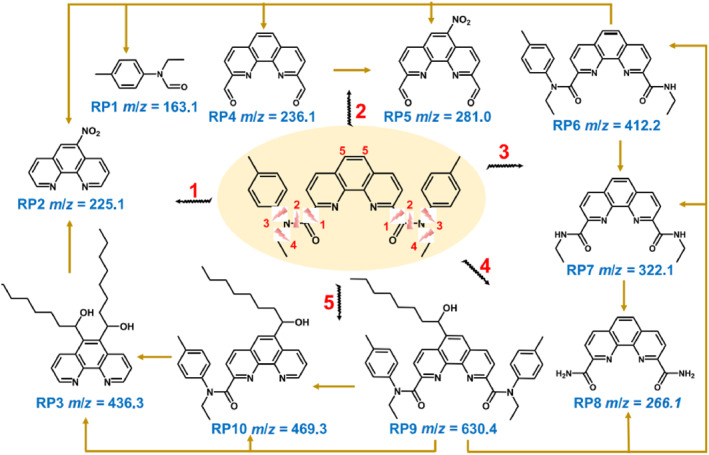
Formulated structure of the radiolytic pathway and products of the novel extractant Et‐Tol‐DAPhen.^[78]^

Another strategy involved the development of novel extractants to reduce or eliminate the effect of TBP degradation products (Figure 8). For example, Nakamura et al.^[79]^ used amide as the extractant, and Shukla et al.^[80]^ pioneered the use of sulfoxide and phosphorus‐containing extractants. These extractants were suitable for inhibiting the negative effects of tributyl phosphate degradation products and preventing easy complexation of fission products. Moreover, the above extractants were difficult to degrade, while the products were easy to remove after degradation. In addition, they only exhibited weak binding ability with the metal ions generated by fission.

### 5.3 Health and environmental considerations

In addition to affecting the process performance, the degradation products of TBP, such as HDBP and H_2_MBP, may pose potential environmental and health issues. These acidic organic phosphates have moderate persistence in aqueous solutions and can form stable complexes with heavy metals and radioactive nuclides (such as Pu^4+^, Zr^4+^), thereby increasing their solubility and potential mobility in the environment. If not handled properly, these complexes may increase the leaching risk of radioactive nuclides in the waste stream, thereby posing long‐term pollution risks to groundwater and ecosystems. Moreover, although TBP itself has relatively low acute toxicity, some degradation products—such as butyl nitrate and carboxylic acids—may have irritant or unknown chronic effects, so they need to be handled with caution during solvent recovery and waste treatment operations. Therefore, effective sealing, degradation monitoring, and final destruction of waste solvents are not only crucial for process efficiency, but also extremely important for minimizing radioactive and chemical hazards.

## 6 CHALLENGES AND FUTURE DIRECTIONS

Although the TBP solvent system in the PUREX process has been widely utilized for spent fuel reprocessing, it still suffers from several challenges. First, it was difficult to prevent TBP degradation under strong‐radiation, high‐temperature, and high‐acidity conditions. Second, degradation products such as HDBP, H_2_MBP and other acid phosphate derivatives would accumulate, resulting in reduced efficiency of uranium‐plutonium recovery and accelerated formation of insoluble complexes with actinides and fission products. This may induce third‐phase formation and equipment corrosion. Third, the anti‐irradiation performance and chemical stability of existing diluents (e.g., kerosene, n‐dodecane) still need to be improved. During long‐term operation, these diluents were prone to nitrification and oxidation, thus negatively affecting the process stability. Although diluent modification (e.g., HOK and hyperbranched alkanes) and process optimization (e.g., controlling the acidity and adding stabilizers) were capable of mitigating diluent degradation to a certain extent, the above strategies remained not suitable for industrial applications. Simultaneously, gaining intensive insights into the above degradation process remained challenging. The simulation accuracy of existing computational models for complex irradiation reactions (e.g., free‐radical chain reactions) was insufficient, and these models did not accurately describe the behavior of multiphase interfaces. In addition, technologies for the on‐line monitoring and real‐time control of degradation products are still immature, implying that dynamic optimization of process parameters remains difficult. Therefore, future research should be focused on the following aspects: First, new extractants (e.g., amides and sulfoxide compounds) and new diluents (e.g., hyperbranched alkanes and ionic liquids) with high stability and low degradability should be developed to enhance the stability of solvent upon the exposure to irradiation and chemical degradation. Second, the interaction mechanism between degradation products and metal ions should be elucidated, and relevant strategies for blocking third‐phase formation through molecular design or the use of additives (such as complex inhibitors) should be developed. This could be achieved through combining with theoretical calculations such as density functional theory and molecular dynamics simulations. Third, advanced characterization techniques (such as in situ spectroscopy and MS) should be developed to achieve real‐time monitoring of degradation products. Fourth, new solvent systems taking into account the requirements for efficient extraction and non‐proliferation were urgently to be developed.

## AUTHOR CONTRIBUTIONS

**Tian Lan**: Conceptualization, writing—original draft, methodology, investigation, data curation. **Jiaxin Liu**: Writing—review and editing; supervision; methodology. **Yi Liu**: Writing—review and editing; validation; supervision; methodology; investigation; funding acquisition; conceptualization.

## CONFLICT OF INTEREST STATEMENT

The authors declare no conflicts of interest.

## Data Availability

Research data are not shared.
